# A Systematic Review of Drug-Loaded Electrospun Nanofiber-Based Ophthalmic Inserts

**DOI:** 10.3390/pharmaceutics13101637

**Published:** 2021-10-08

**Authors:** Safaa Omer, Romána Zelkó

**Affiliations:** University Pharmacy Department of Pharmacy Administration, Semmelweis University, 1092 Budapest, Hungary; safaa.omer@phd.semmelweis.hu

**Keywords:** ophthalmic inserts, drug-loaded, nanofibers, electrospinning

## Abstract

Currently, ocular inserts and nanoparticles have received much attention due to the limited bioavailability of conventional eye preparations and the toxicity problems of systemic drug administration. The current systematic review aims to present recent studies on the use of electrospun nanofiber-based ocular inserts to improve the bioavailability of drugs used for different ophthalmic diseases. A systematic search was performed in PubMed, Ovid Medline, Web of Science, ScienceDirect, Scopus, Reaxys, Google Scholar, and Google Patents/Espacenet taking “drug-loaded”, “nanofibers”, and “ophthalmic inserts” and their equivalent terms as keywords. The search was limited to original and peer-reviewed studies published in 2011–2021 in English language. Only 13 out of 795 articles and 15 out of 197 patents were included. All results revealed the success of nanofiber-based ocular inserts in targeting and improved bioavailability. Ocular inserts based on nanofibers can be used as safe, efficient carriers for the treatment of anterior and posterior eye diseases.

## 1. Introduction

**Figure 1:**
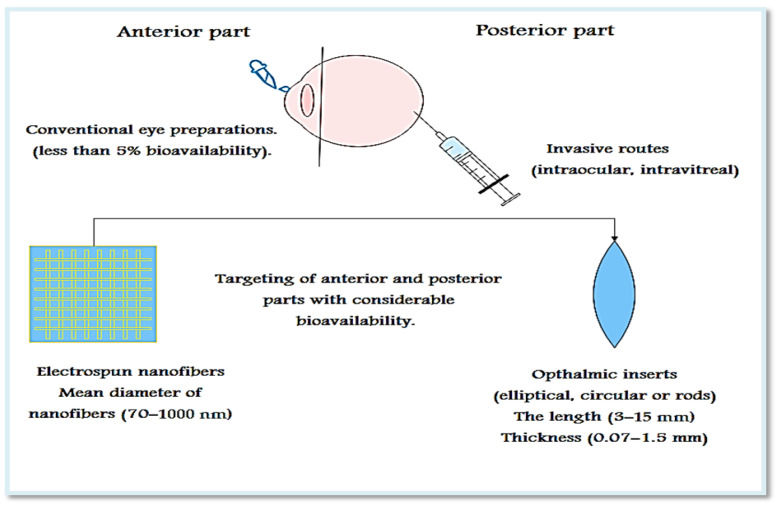
The most common routes of targeting different parts of the eye.

The unique anatomy and physiology of the eye, because of the physical and blood barriers, makes the targeting of ocular diseases a very difficult and challenging process [1,2]. These barriers interfere with drug absorption and diminish the drug concentration in the eye due to dilution and early drainage through lacrimation and poor permeation due to the effect of blood-retinal and blood-aqueous barriers [3,4,5]. Drugs used for the treatment of ocular disorders are administered via different routes (Figure 1): systemic, topical, subconjunctival, intrastromal, intracameral, intrascleral, intravitreal, suprachoroidal, and subretinal [6,7]. Oral administration of most drugs is subjected to kidney clearance, hepatic clearance, and failure of absorption due to physical and blood barriers. Likewise, absorption of drugs with the use of parenteral administration may subjected to retardation by blood-aqueous and blood-retinal barriers [8,9]. The most frequently used method is administration of drugs through the conventional topical route, which is characterized by being non-invasive, easy to self-administer, and more acceptable to patients. Nevertheless, it can only be used for the treatment of diseases affecting the surface and anterior segment of the eye, because it has poor bioavailability (less than about 5% of the drug is retained on the ocular surface). In addition, this method is not suitable for hydrophobic drugs or drugs that are unstable at a pH level tolerable to the eye and require frequent administration in order to overcome the barriers and nasolacrimal duct drainage [10,11]. Suitable for administration of up to 500 μL and relatively less invasive, subconjunctival administration is a highly convenient route and can be successfully used for targeting anterior or posterior segments [6,7]. Direct administration of the drug to the site of action with lower bioavailability can be done invasively based on intraocular routes [6], with intravitreal injection being the most promising for targeting posterior diseases, but it can be accompanied by retinal detachment, increased intraocular pressure, intravitreal hemorrhage, cataract, or endophthalmitis, which results in poor patient adherence [2,12].

Apart from the conventional and parenteral routes, many alternatives, such as permeation enhancers, viscosity modifiers, pro-drug strategies, nanoparticles, long-term or permanent punctal inserts, corneal shields, contact lenses, mini-tablets, disposable lenses, biodegradable polymer systems, hydrogels, implants, and inserts, have been investigated to overcome the ocular barrier problem and increase bioavailability [12,13,14,15,16]. They act by increasing the contact time and slowing down the elimination of the drug from the eye [14]. A very promising approach for ocular targeting is the use of novel drug delivery systems based on nanotechnology; consisting of colloidal particles with sizes ranging from 1–1000 nm [17], nano-carrier drug delivery systems easily penetrate different barriers and have a lower chance of causing eye irritation. Moreover, they offer targeted and sustained release effects [18,19]. They include, but are not limited to, nanocapsules, liposomes, nanomicelles, lipid nanoparticles, niosomes, dendrimers, nanosuspensions, nanoemulsions, and nanocrystals [20,21].

Among the novel alternative approaches, ophthalmic inserts are sterile solid or semi-solid devices consisting of polymeric material with or without medicament, whose suitable size and shape are designed to be placed into the conjunctival sac [22]. Ocular inserts have received attention, as they increase the residence time of the drug on the eye surface, allow slow and controlled drug release, and reduce the overall dose and dose frequency. Moreover, they are stable and eliminate the need for preservatives, thereby reducing the possible side effects and increasing the shelf life of the drug compared to liquid formulations. They are classified into soluble, insoluble, and bio-erodible inserts. Soluble and erodible inserts undergo slow dissolution and do not need to be removed, and insoluble inserts should be removed from the eye when they are free of drugs [23].

**Figure 2:**
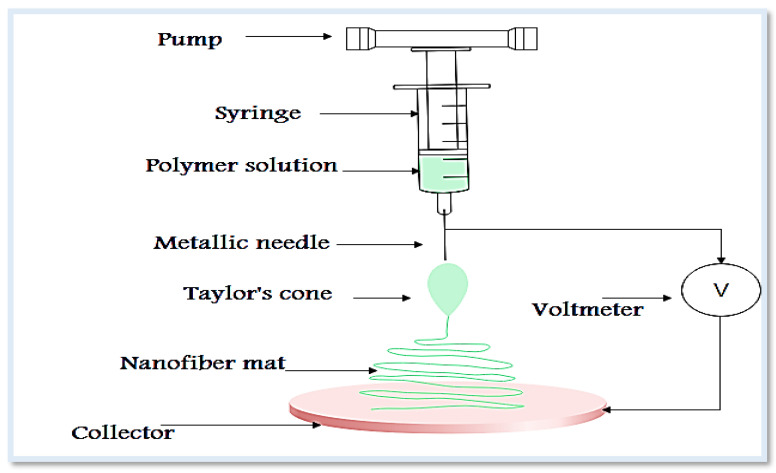
Schematic diagram of electrospinning process.

Drug release from ocular inserts takes place by three mechanisms: diffusion, osmosis, and erosion. Ocular inserts are prepared using solvent casting, glass substrate, and melt extrusion [24] techniques, and electrospinning (Figure 2) has been used as a simple, versatile technique for the production of nanofibrous film with unique properties [25]. The process of electrospinning is highly flexible and provides the chance to fabricate a wide range of natural and synthetic polymers or drugs; the technique relies on the application of a high voltage between the metallic needle and a grounded collector [26,27]. When the applied voltage exceeds certain critical value, the liquid is ejected from the needle, forming a conical droplet known as Taylor’s cone, followed by elongation, thinning and precipitation on the surface of the collector [27]. The resulted fibers are randomly deposited, but for more structured and aligned fibers rotating mandrel or a wheel-like collector are used [28]. The electrospun nanofibers with diameters of 100 nm [29] are constructed from a wide range of biodegradable and biocompatible polymers. The process is carried out simply by applying a high voltage to a drug or mixture of a drug and polymer solution or melts. The drugs can be incorporated through different methods such as blending, surface immobilization, and emulsion [28]. A wide range of drug materials can be encapsulated ranging from small inorganic molecule to large molecules of biological drugs such as protein and nucleic acids. In addition, the technique enables delivery of more than one drug in one step. The produced fibers have unique properties making them a suitable candidate for variety of pharmaceutical and biomedical [26,28,30]. Their large surface area enables ease of surface engineering and modification using different materials, such as permeation enhancers and mucoadhesive agents, and with the proper polymer selection, systems with controlled, sustained, and targeted release can be produced [31,32,33]. Furthermore, the produced fibers are homogeneous and highly reproducible [34], and due to their characteristic size and texture, they also represent good candidates for tissue reconstitution and promotion of human astrocytoma cell growth [35] and cell migration and proliferation [36]. A wide range of polymers are used for the fabrication of nanofibers for ocular uses, they are classified as natural, semisynthetic, and synthetic [37]. The final properties and quality of fibers are determined by many factors of which the type and concentration of polymers are of paramount importance. Some of the suitable polymers for the electro-spinning technique include polyesters (e.g., poly-glycolic acid- (PGA), poly-lactic acid- (PLA), polycaprolactone- (PCL)) [33]. These polymers are characterized by possessing sufficient mechanical strength to be electrospun, biocompatible, and useful for cell adhesion [34]. In some cases, a blend of the polymers is used together to control the drug release. Other cases include surface modification such as coating with mucoadhesive polymers or complexation with solubility or permeability enhancers [33]. According to the general rule, like dissolves like, hydrophilic polymers are a suitable candidate to encapsulate hydrophilic drugs without sustaining the release, while hydrophobic polymers can modulate sustained effects [37]. Natural polymers are widely used for biomedical applications [38], but synthetic polymers are used to fabricate products with superior quality; therefore, a combination of polymers from different origins is more advantageous for added functional properties [37]. Chitosan is a polysaccharide with a cationic amino group with wide pharmaceutical and biomedical applications [39]. The presence of an amino group leads to extensive protonation in aqueous media due to electrostatic interaction with a solvent molecule, which alters the solubility, viscosity, and mucoadhesive properties of the polymer [40]. Chitosan has gained significant attention in ophthalmic formulations due to its mucoadhesive properties, biocompatibility, and biodegradability and can increase drug permeability [19,41,42]. Nevertheless, it has several limitations include low stability in acidic media and poor mechanical strength. Complexation with cyclodextrins has been developed to strengthen the chitosan structure [43]. Another polymer relatively similar to chitosan is hyaluronic acid; it is a mucoadhesive polymer, forming a hydrogen bond with mucin; hence, it can successfully be used to prolong the drug release rate [37]. Gelatin as biopolymer is a natural protein of an animal origin that contains arginine-glycine-aspartic acids and metalloproteinase residues. Being widely available with lower antigenicity, possessing good bioadhesive properties, and promoting tissue regeneration renders it a good candidate for several biomedical and drug delivery applications. However, it has poor aqueous stability and low mechanical strength [44]. Integration of carbon nanotubes, graphene oxide, and carbon nano-onion into gelatin base improves mechanical strength and enables surface modification [45]. The drug loading and release kinetics are affected by molecular weight and cross-linking of the gelatin [46]. Poly-caprolactone (PCL) is a semi-crystalline hydrophobic biopolymer characterized by being biocompatible, possess significant toughness, soluble in most organic solvents; therefore, it is suitable for biomedical and drug delivery purposes [33]. In addition, it has a slow degradation rate, thereby can extend the drug release [25]. However, PCL has some limitations, such as insufficient strength, which necessitates additional reinforcement, such as integrating carbon nano-onions [47,48]. Bovine serum albumin (BSA) can be used as a nanocarrier for various drug molecules, incorporated through electrostatic interactions and covalent or non-covalent conjugation. It has many advantages, including being affordable, producing no immunogenic response, biodegradable, and modulating for different release patterns. Although it is very difficult to produce electrospun nanofibers from BSA alone, they can be combined with other polymers such as polyvinyl alcohol (PVA) and polyethylene oxide (PEO) [49]. Zein is a water-insoluble plant protein that has been used in drug delivery and coating. It requires physical and chemical treatment to improve its low mechanical strength and increase its water stability. Poly 4-mercaptophenyl methacrylate-carbon nano-onions can be incorporated within zein for enforcement of the mechanical strength [50].

Although there are many published reviews describing ocular inserts, few of them cover the formulation of ocular inserts based on nanofibers; therefore, the current systematic review aims to describe the use of ocular inserts in the treatment of various eye diseases by shedding light on nanofiber-based ocular inserts as a promising, non-invasive method for targeting the posterior segment of the eye and improving bioavailability.

## 2. Materials and Methods

Preferred Reporting Items for Systematic Reviews and Meta-Analyses (PRISMA 2020) guidelines were followed to search for the relevant studies and report construction.

### 2.1. Eligibility Criteria

The following criteria were set for articles to be eligible for inclusion in this systematic review: Original research studies published in peer-reviewed journals and published patents were included, while review articles, conference papers, editorials, and commentaries were not. The study was limited to articles published in 2011–2021 in the English language. In addition, only ocular inserts or products to be placed in the conjunctival sac or cul-de-sac were considered, and all other dosage forms, including contact lenses and ocular implants, were excluded. The study included only nanofibrous ocular inserts fabricated via electrospinning technique; other techniques for fiber production were not considered. No clinical trial limits were set, and all in vitro and in vivo studies were eligible. Studies comparing electrospinning with other techniques were also included.

### 2.2. Search Strategy

To find the relevant articles and patents of drug-loaded electrospun nanofibrous ocular inserts, a systematized search was performed in PubMed, Ovid Medline, Web of Science, ScienceDirect, Scopus, Reaxys, Google Scholar, Google Patents, and Espacenet using the specified keywords together with their equivalent synonyms. We built up our search queries as: (Drug-loaded) AND (electrospinning) AND (Nanofibers OR nanofibrous) AND (ocular inserts OR ophthalmic inserts). The results were synthesized and tabulated accordingly.

### 2.3. Data Collection and Extraction

We used a PRISMA 2020 flow diagram to extract the most relevant data essential for synthesizing the results. First, all results obtained from all databases were exported to the Mendeley reference manager, and duplicate studies were removed. The rest of the articles underwent two successive screening processes: Irrelevant articles based on title were immediately excluded, then the abstracts and full texts of eligible articles were reviewed and analyzed. The relevant articles were double-checked by the reviewers based on the inclusion criteria, and the required information was extracted and tabulated into the following variables: polymer base, loaded drug/concentration, dimensions of inserts used in the study, diameter of nanofibers, in vivo animal model, and effects/properties of presented system.

## 3. Results

### 3.1. Database Search and Included Studies

**Figure 3:**
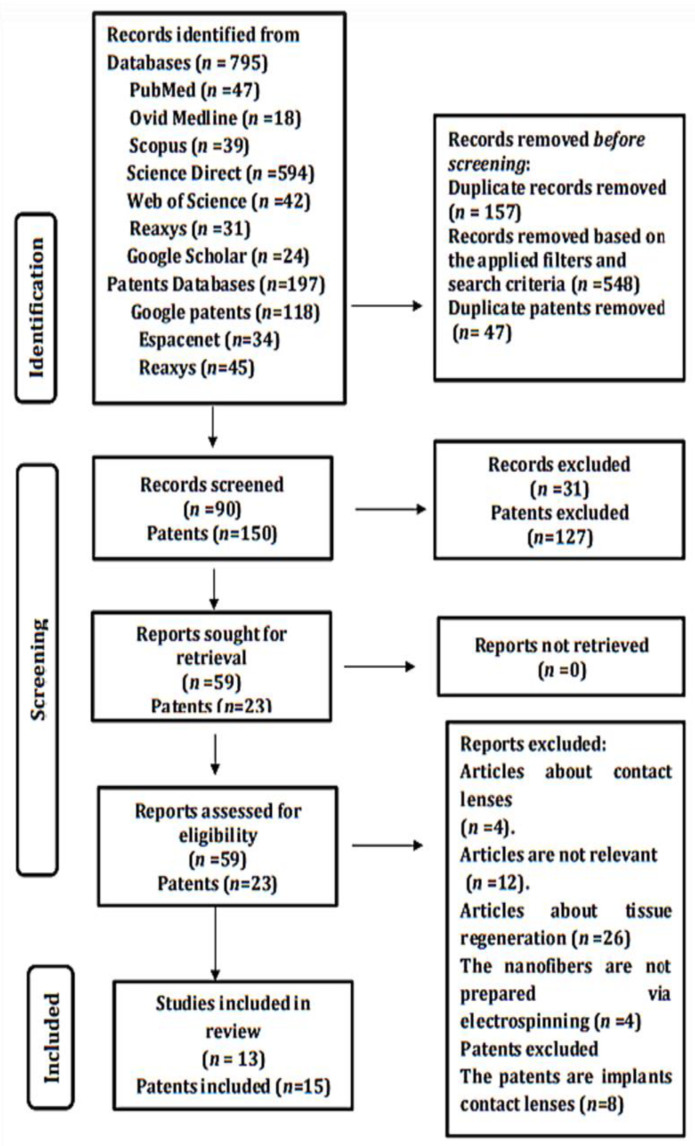
PRISMA-2020 flow diagram showing relevant articles and patents included in the study.

A total of 795 articles and 197 patents were obtained from all database searches, among which 594 were from Science Direct, 47 from PubMed, 42 from Web of Science, 39 from Scopus, 31 from Reaxys, 24 from Google Scholar, and 18 from Ovid Medline. Of the total patents obtained, 118 were from Google Patents, 34 from Espacenet, and 45 from Reaxys. Only 13 articles and 15 patents were included in the review, and they were selected based on the specified inclusion criteria. The process of identification and screening is presented in Figure 3.

### 3.2. Results of Studies

According to the specified criteria, all relevant articles were thoroughly reviewed, and are summarized in Table 1. According to the extracted relevant information, New Zealand albino rabbits and some in vitro models are used to study eye toxicity, and the results revealed no or lower eye toxicity. The main focus was on increased residence time and bioavailability. The results showed the possibility of modulating the drug release from a few minutes up to a month based on the polymer base used and the properties of nanofibers, and the majority of studies showed controlled drug release. The formulations were compatible with the eye and the particle size showed no signs of ocular irritation.

Here is a summary of the published studies based on the use of electrospinning technology for the fabrication of nanofiber-based ocular inserts and formulations designed to be used in the cul-de-sac: Besifloxacin HCl loaded inserts were prepared by electrospinning and investigated in in vitro, ex vivo, and in vivo studies as a potential alternative to commercial formulations. The formulations were subjected to modification with the addition of mucoadhesive polymer (sodium alginate (SA) or thiolated sodium alginate (TSA) as coating material) or corneal permeation enhancer (HP-β-CD) to the base polymer system of poly-caprolactone (PCL) and polyethylene glycol (PEG) in a 2:1 ratio of PCL/PEG. The in vitro studies, including thickness, diameter, degradation, and encapsulation, were within acceptable limits. The studies showed that the formulations produced no cytotoxic effect, with notable activity against bacteria. Corneal keratitis was significantly reduced by treatment with TSA-coated and HP-β-CD drug complex inserts [33].

Core-shell electrospinning was successfully used to fabricate a nanofibrous mat of pirfenidone-PLGA/moxifloxacin-PVP for the treatment of corneal abrasion. The results showed non-porous and smooth fibers with a diameter of 630 ± 220 nm that were capable of sustaining the release of both drugs and suitable for once-daily use [36]. The same results from pharmacokinetics studies showed the possibility of extended release over 24 h, while a Draize test showed mild irritation to the eye. Furthermore, antimicrobial and anti-scarring effects obtained were comparable to those of free solution [51].

Electrospun nanofiber inserts were fabricated and compared to inserts prepared by solvent casting employing poly-lactic acid (PLA) and poly-vinyl alcohol (PVA) as the polymeric system. The results showed thin, uniform inserts of nanometer size. In addition, the drug content was found to be more uniform in the inserts prepared by electrospinning, and it was concluded that electrospun nanofiber inserts could be a potential alternative to conventional eye drops [52]. Electrospun nanofibers loaded with gentamicin and methylprednisolone were prepared in different structures (single-jet electrospinning, sandwich structure using single-jet electrospinning, and core-shell electrospinning). All formulations were evaluated for physicochemical and antibacterial properties against *S. aureus*. The formulations showed acceptable mechanical and antimicrobial activity, with diameters of 70–350 nm, and the core-shell preparation showed the best drug release [53].

New nanofiber-based ocular inserts were prepared using hyaluronan (HA) and polyvinylpyrrolidone (PVP) for dual delivery of ferulic acid (FA) as antioxidant and ε-polylysine (ε-PL), an antimicrobial peptide. Two series of inserts were prepared, blank, and FA-loaded. The prepared inserts were subjected to physical, morphological, compatibility, release, and antimicrobial studies. The results showed acceptable thickness of 270 ± 21 μm to 273 ± 41 μm with fiber diameter of approx. 100 nm to 1 μm; in addition, they demonstrated adequate drug release, with antimicrobial activity against *Pseudomonas aeruginosa* and *Staphylococcus aureus* [54].

Ocular polymeric inserts loaded with triamcinolone acetonide (TA) have been developed in two steps: electrospinning of a solution of poly-butylene succinate (PBS), followed by surface modification through plasma activation and reaction with inulin, heparin, and α,β-poly(*N*-2-hydroxyethyl)-d,l-aspartamide. The morphological results showed a flexible non-porous scaffold with fibers ranging from 1 to 3 μm in diameter. The surface modification allowed stable, non-erodible, mucoadhesive, and highly loaded inserts that were compatible with human cells; it also allowed extended drug release for up to 30 days [34]. Nanofiber inserts loaded with azithromycin were prepared using a mixture of chitosan, polyvinyl alcohol, and polyvinyl pyrrolidone via electrospinning. The formulated inserts were subjected to physicochemical, morphological, in vitro, and in vivo release, antibacterial activity, cytotoxicity, and ocular irritation evaluation. The results showed acceptable hardness and uniform weight and thickness, with fiber diameter ranging from 119 ± 29 to 171 ± 39 nm. The inserts were also found to be nontoxic and non-irritating to the rabbits’ eyes. The drug release was extended up to 6–8 days [55].

In an attempt to target retinal inflammatory disease, nanofibrous ocular inserts loaded with fluocinolone acetonide were prepared and evaluated for permeability, in vivo pharmacokinetic, and in vivo release as well as other mechanical and chemical characteristics. Preclinical results revealed the possibility of retinal delivery with no cytotoxicity. The obtained fibers were smooth, non-woven, and homogeneous. Degradation and release studies confirmed extended drug release up to 12 days [25].

Nanoparticles loaded with azithromycin were incorporated into electrospun nanofibers to obtain ocular inserts that were mucoadhesive and biodegradable. The formulations were characterized in vitro, ex vivo, and in vivo. The results showed improved bioavailability, low risk of toxicity, and prolonged drug release over 10 days [56]. Two polymer series, polycaprolactone (PCL) and polyvinyl alcohol (PVA), were used to formulate biodegradable polymeric patches containing timolol maleate and dorzolamide hydrochloride for cul-de-sac insertion. The formulations were characterized in terms of morphology, folding endurance, drug release, ocular irritation, and in vivo efficacy. The fibers were uniform and smooth with sufficient mechanical strength. An in vitro release study for up to 24 h confirmed a single daily dose. Ocular irritation results showed minor irritation with PCL formulation and no comparable effect with PVA patches. The products successfully reduced the induced intraocular pressure and maintained for up to 72 h [57].

An internal layer of hydrophilic chitosan/polyvinyl alcohol (CS/PVA) and an outer layer of hydrophobic Eudragit RL100 were used in the fabrication of ocular inserts for delivery of ofloxacin (OFX) to increase the residence time on the eye. All parameters related to strength, thickness, and morphology were within acceptable limits. The formulations showed significant in vitro antimicrobial activity against *S. aureus* and *E. coli.* The formulations allowed prolonged release for up to 95 h with no signs of ocular irritation as demonstrated by in vivo studies [58]. Four formulations were used to prepare nanofibrous ocular inserts; three of them were chitosan-based and the fourth was composed of Eudragit S100 and Zein for sustained delivery of triamcinolone acetonide. All fibers were smooth and fibrous except the formulation containing a mixture of PVP, PVA, and chitosan. In vitro release studies demonstrated sustained drug release (zero-order rate), and no in vivo studies have been done [38].

Based on the results summarized in Table 1, it is obvious that the formulation of these ocular inserts depends on the use of natural and/or synthetic polymers. These polymers are involved in determining the final quality of products. The most widely used polymers include polyglycolic acid (PGA), polylactic acid (PLA), polycaprolactone (PCL), polyvinyl alcohol (PVA), polyacrylic acid (PAA), polyvinyl pyrrolidone, hyaluronic acid, chitosan (CS), polyethylene oxide (PEO), polymethacrylate, and cellulose derivates [25,33,37,41]. The majority of these polymers are biodegradable and classified as being soluble, insoluble, or bioerodible. With the proper base selection, a wide range of ocular inserts with different forms of drug release and targeting can be obtained. The results of many studies based on polymer type are given in Table 2.

### 3.3. Ocular Insert Patents Studies (2011–2021)

Ocular inserts have recently received attention and many patents have been published demonstrating conventional and nanofiber-based formulations. Some examples of interesting and promising systems are presented here along with summaries, shown in Table 3. An Australian patent describes a nanostructured biocompatible wafer for placement in the conjunctival cul-de-sac. The wafer contains a tissue-reactive mucoadhesive polymer, and provides a method for treating glaucoma or infection on the eye surface. The thickness of the wafer is between 0.05 and 0.5 mm, preferably between 0.05 and 0.1 mm, but it is not limited to hydrophobic polymers or any combination of biodegradable polymers, with the polymers available commercially and approved for human use being the best choice [75].

Another patent describes an ocular delivery system that consists of a nanofibrous matrix containing drug-loaded nanoparticles as a candidate for ocular inserts. In addition, the method of preparation (electrospinning) and medical uses of the nanofibrous ocular system are demonstrated, which include anti-microbial, anti-glaucoma, anti-inflammatory, analgesic, anesthetic, or combined effects. Biodegradable hydrophobic polymer and/or biodegradable amphiphilic polymer can be used. The system can also contain a mucoadhesive polymer to provide controlled release of drug over a period of at least 3 days [76].

Another patent describes various shapes of ocular inserts together with methods of preparation and potential uses. Hydrophilic polymers with biodegradable, bioabsorbable, or bioerodible properties are used. In addition to active ingredients, it may contain dyes, lubricant, emollient, and jelling agent. The system can be loaded with thermo-labile, poorly soluble, soluble, micronized, and nanoparticle substances. The pharmaceutically active agents include anti-bacterial, steroidal and non-steroidal anti-inflammatory, anti-allergy, anti-viral, anti-cholinergic, and mydriatic drugs, or any suitable combination [77]. Another patent discusses the bimatoprost composition, preparation, and devices comprising these compositions. These formulations are responsible for sustained release of bimatoprost to the eye. The composition of this invention comprises stable amorphous bimatoprost with a thermoplastic polymer matrix, such as acrylonitrile butadiene styrene (ABS), celluloid, cellulose acetate, ethylene-vinyl acetate (EVA), ethylene vinyl alcohol (EVOH), polyacrylate (acrylic), or polyacrylonitrile (PAN or acrylonitrile). It may contain thermosetting polymers. This medical device can serve as an ocular insert with a ring shape (ring diameter can be about 10 to 40 mm or about 20 to 30 mm) [78].

A patent describes a method for preparing rifampicin film to overcome uneven drug content by using poly-vinyl alcohol (PVA) via a simple method with temperature, pressure, and time control [79]. The patent pertains to ocular inserts for sustained release of steroids to the eye. The steroids are considered as articles from D1-L-D2 (A-I), where D1 and D2 are steroid radicals and L is linker that is covalent to D1 and D2. The article can be fiber, fiber mesh, nanoparticles, microparticles, or woven or non-woven fabric. The linker can be carbonate or carbamate ester [80].

Another patent describes soft polymeric hydrogel ocular inserts that are readily available and comfortable for use to release drug and lubricant to the anterior and posterior segments of the eye in a controlled manner. The hydrogel material is derived from at least one arylborono-containing hydrophilic copolymer and at least one mucoadhesive polymer, and cyclic boronic ester. The patent also describes the method for preparation [81]. Another patent describes atropine sulfate ocular inserts and the method of preparation to enhance stability and bioavailability by utilizing hydroxypropyl methylcellulose, polyvinylpyrrolidone, sodium carboxymethyl cellulose, and gelatin or any suitable combination [82]. Another patent describes the delivery of at least one drug to the desired site of action of the human or animal eye. The system contains at least two polymers, preferably from the class of polyethylene oxide block copolymers and cellulose derived polymers, such as hydroxpropyl cellulose. It may also contain an anti-collapsing agent, such as amino acid. The active ingredients include prostaglandin, beta blockers, alpha agonists, carbonic anhydrous inhibitors, or any suitable combination [83].

Another patent describes polymeric ocular inserts composed of a pharmaceutically active semi-crystalline or crystalline agent dispersed in a polymer matrix in order to provide a formulation that is more stable and has fewer impurities. The method of preparation is also described. The composition includes for example, bimatoprost as a pharmaceutically active ingredient, a polymeric matrix, which is a thermoplastic polymer such as acrylonitrile butadiene styrene, and a thermosetting polymer such as silicone and polyesters [84].

Another patent describes an ocular insert that is considered as a new biocompatible polymer-based controlled drug delivery system for the release of suitable drug to the eye for up to 300 days. The inserts are prepared with different shapes and are suitable for self-administration, and can be inserted in the lower or upper fornix conjunctiva. At least one drug can be incorporated, including antibiotics, antibacterials such as sulfonamides, antivirals, anti-allergy, anti-inflammatories such as hydrocortisone or hydrocortisone acetate, decongestants such as tetrahydrozoline, miotics and anticholinesterase, sympathomimetics such as epinephrine, immunological drugs such as vaccines and immune stimulants, hormonal agents, growth factors, or carbonic anhydrase inhibitors. The polymers used are polycaprolactone (PCL), polyethylene glycol (PEG), or co-polymers PEG-PCL, or a mixture of these [85].

Another intervention aims to provide voriconazole-loaded ocular film for continuous release of the drug. The ocular film consists of nanopolymer fiber and film-forming and other excipients. Electrospinning is used to prepare the film in order to obtain nanopolymer fibers with uniform morphology. Polyvinyl alcohol, acrylic resin, polyvinylpyrrolidone, polyvinylpyrrolidone derivative, cellulose, cellulose derivative, and chitosan individually or in combination are considered to be the best materials for film preparation [86]. Another patent describes dissolvable polymeric eye inserts with a biodegradable polymer for sustained release of drugs and lubricants to the anterior and posterior segments of the eye. They provide a way to treat different eye disorders by incorporating different active pharmaceutical ingredients. The inserts are comfortable for patients and the thickness of the film ranges from 50–250 μm, and preferably from 70–150 μm. The polymers used include hyaluronic acid, hydroxypropyl guar (HP guar), and a plasticizer, such as polyethylene glycol (PEG). The inserts are suitable for insertion in the lower eyelid. The composition is rapidly wetted by tears to release the lubricant. The active ingredients may be added to the same polymeric base [87,88,89].

## 4. Discussion

It is a very difficult process to deliver drugs to the eye, especially the posterior segment, due to the presence of physical barriers as well as blood retinal barriers, particularly when using conventional topical formulations. Therefore, scientists have performed many studies on eye diseases in an attempt to deliver drugs to the target site of action with sufficient bioavailability. Nanotechnology-based drug carriers have been investigated for their potential technological and therapeutic advantages for ocular delivery. Nanocarriers are used to deliver drugs for local or systemic effect by being localized to a specific site in the eye and releasing the required drug concentration through diffusion or as a response to external stimuli [18,90]. They offer numerous advantages; for example, the surface of the nanocarrier can be modified using different polymers for different purposes, such as mucoadhesive properties, by increasing the residence time, thus tailoring the drug release to reach controlled or sustained delivery. Furthermore, enhanced bioavailability with a lower risk of adverse effects and eye irritation can be obtained to increase patients’ adherence to medications [28,91,92,93,94,95].

The current systematic review demonstrates the efficiency and superiority of nanoformulation, particularly nanofibrous webs, as a promising alternative to conventional methods regarding site targeting and improved bioavailability. All toxicity studies proved their safety in animal models and in vitro alternative models. Pharmacokinetic studies have shown sustained drug release through different models such as zero-order and Higuchi models, which allows the possibility for single-dose administration for up to 30 days [34]. Combining the advantages of nanofibers with the advantages of ocular inserts will increase the bioavailability by many times [58] and reduce or even eliminate the disadvantages of using normal macro-size ocular inserts.

## 5. Future Perspective

Many people have ocular diseases, which interfere with quality of life, and the number is increasing by 7 million per year [96]. Even though many published studies have demonstrated that ocular inserts are a successful and non-invasive method of delivering drugs to the eye, only a small number of ocular inserts are available on the market, for example, Lacrisert^®^, a topical insert approved by the U.S. Food and Drug Administration [64] and Mydriasert© [97]. A significant amount of research on tailoring the delivery of various drugs to different parts of the eye using ocular inserts has provided very promising results, such as delivery of antimicrobial peptides to the pre-corneal area [98] and ocular inserts for delivery of thermolabile therapeutics [71].

Since nanotechnology has received a great deal of attention in recent years, nanofibers are expected to become an integral part of frequently used dosage forms in the near future, as they are able to penetrate and target different sites, including posterior segments of the eye [38] and the vitreous cavity for the treatment of retinal diseases [99]. From a technical point of view, nanofibers can be used directly as films [58] or after further processing into suitable ocular inserts or other surface modifications [100]. In addition, nanofibers can be formulated using a simple, versatile electrospinning technique [31]. The process enables encapsulation of more than one drug in one step [38], which will result in decreased multiple-drug regimens and increased patient compliance [27,101]. It also enables encapsulation and delivery of macromolecules such as genes and proteins, and this will open the door for research in this area, especially since there are not many studies on macromolecules [102]. In addition it can be scaled up for large-scale production to meet the requirements and sustainability of pharmaceutical companies [103].

Obviously, ocular inserts have also been intensively investigated because of their unique advantages as an alternative way to target eye disease, considering the possibility of fabricating them using different methods and polymeric materials. To meet the needs of the patients and minimize their suffering, a new non-invasive drug delivery system that offers good bioavailability and has the potential to deliver medicaments to posterior parts of the eye is expected to be investigated and introduced to the market in the near future. As many studies have proven the success of ocular inserts, the application of electrospun nanofibers as film or as part of ocular inserts is expected to result in a great advancement in the targeting of eye diseases, if these studies find a place in human clinical trials to confirm their efficiency and nontoxicity.

## 6. Conclusions

Eye problems are increasing daily; therefore, a smart approach is needed. It has been proven that drug delivery to the eye involves many problems that result from the barriers present in the eye, including the retinal blood barrier, lacrimation, eye blinking, and dilution, which is eventually reflected in poor drug bioavailability, particularly when using conventional topical drug delivery systems such as eye drops. In order to overcome such barriers, it is important to increase the contact time of the eye formulation to increase absorption and decrease the frequency of drug administration.

Different unconventional approaches are available, but every approach comes with some limitations. Nanofibers have gained attention recently, since they have superior advantages compared to other available systems; they are less irritating to the eye due to their small size and can be fabricated using different polymeric systems, therefore sustained release formulations could be applied, resulting in lower administration frequency. In addition, many studies have demonstrated successful posterior eye targeting.

An interesting characteristic of nanoparticulates is their large surface area, which allows the fabrication of different structures, including ocular inserts. Ocular inserts have been found to have many advantages: they provide increased bioavailability through the prolonged residence time of the drug on the conjunctival surface, can be formulated using different polymeric materials and methods, and allow the preparation of preservative-free formulations, thus resulting in lower eye sensitivity or even no sensitivity or irritation. In the light of the advantages of nanofibers and ocular inserts, a synergistic effect could be obtained by formulating ocular inserts with a nanofibrous architecture. Many published studies confirm the possibility of using such systems as an alternative to conventional topical formulations with better results.

## Figures and Tables

**Table 1:** Summary of nanofiber-based ocular insert studies.

No	Polymer Base	Loaded Drug/Concentration	Dimensions of Inserts Used	Diameter of Nanofibers	In Vivo/Animal Model	Effects/Properties	References
1	Polycaprolactone (PCL), polyethylene glycol (PEG), sodium alginate (SA), thiolated sodium alginate (TSA)	Besiloxacin HCl (BH) (40 μg per 1 cm^2^)	3.5 mm^2^ (thickness: 0.66 ± 0.004; diameter: 6.7 ± 0.012)	Less than 1057 nm	Yes, New Zealand albino rabbits	Besifloxacin HCl loaded inserts were developed and investigated in vitro, ex vivo, and in vivo for treatment of bacterial keratitis	[33]
						SA and TSA increased bioadhesion of formulations	
						Inserts showed burst release in first 2 days, followed by slow-release profile	
2	Poly-lactic-co-glycolic acid (PLGA), polyvinylpyrrolidone (PVP)	Moxifloxacin HCl (1% *w*/*v*) pirfenidone (2% *w*/*v*)	0.5 cm × 0.5 cm	Drug-loaded fibers were 630 ± 300 nm	Yes, New Zealand male albino rabbits,	Step 1: Successful fiber preparation with encapsulation of two drugs and sustained drug release	[36,51]
						Step 2: In vivo pharmacokinetic, antimicrobial, and scar healing properties; release rate over 24 h	
3	Polylactic acid (PLA), poly(vinyl alcohol) (PVA)	Dexamethasone (1, 5, and 10% *w*/*w*)	Thickness of fibers ranged from 50 to 93 μm	Within nanometer size	No in vivo studies have been done	Electrospun nanofibrous inserts were fabricated and compared to solvent casting ocular inserts	[52]
						Electrospun nanofibrous inserts showed first-order release rate	
						Results revealed superiority of electrospun nanofibrous inserts over solvent cast inserts	
4	Polycaprolactone, poly (lactic-co-glycolic acid), polyvinyl alcohol	Gentamicin (GNT) (10% *w*/*w*), methylprednisolone (MP) (6% *w*/*w*)	NA	Mean range was 70–650 nm	No in vivo studies have been done	Simple, sandwich, and core-shell nanofibers were prepared and evaluated for dual sustained delivery of gentamicin (GNT) and methylprednisolone (MP)	[53]
						Core-shell formulation showed best release profile	
5	Hyaluronan (HA), polyvinylpyrrolidone (PVP)	Ferulic acid (FA) (5.7 ± 0.2% *w*/*w*)	Mean thickness of 270 ± 21 μm	Approx. 100 nm to 1 μm	No in vivo studies have been done	Development and evaluation of nanofiber inserts for dual release of ferulic acid and ɛ-polylysine (ɛ-PL)	[54]
						Blank inserts released ɛ -PL within 30 min and FA-loaded inserts completely released antioxidant within 20 min	
						All formulations were effective against Pseudomonas aeruginosa and S. aureus	
6	Poly(1,4-butylene succinate) (PBS)	Triamcinolone acetonide (TA) (2 mg/cm^2^)	Scaffold disk: 0.4 cm diameter	Range of 1–3 μm	No in vivo studies have been done	Preparation of novel inserts loaded with triamcinolone acetonide	[34]
						Further modification through plasma-induced chemical functionalization	
						Formulation resulted in sustained drug release (up to 30 days, Higuchi model) with good compatibility with human cells	
7	Chitosan/polyvinyl alcohol/polyvinyl pyrrolidone (CS/PVA-PVP)	Azithromycin (AZM)(10% *w*/*w*)	Diameter: 6 mm; thickness: 0.108 ± 0.012 to 0.121 ± 0.002 mm	Mean range of 119.01 ± 29.77 to 171.61 ± 39.40 nm	Yes, New Zealand rabbits	Nanofiber inserts loaded with azithromycin were prepared	[55]
						Stable	
						Uniform weight and thickness	
						Non-irritating and non-toxic	
						Cross-linked nanofibers enabled more controlled drug release than non-cross-linked	
8	Polycaprolactone (PCL)	Fluocinolone acetonide (1–5% *w*/*w*)	$2\;{\mathrm{cm}}^{2}$	Average range of 350–400 nm	Yes, New Zealand white rabbits	Development and characterization of preservative-free nanofibrous ocular inserts	[25]
						Homogeneous, non-woven nanofibers	
						Burst drug release phase followed by a steady release rate up to 11 days	
						High drug permeation to retina	
						No ocular irritation	
9	Poly(lactic-co-glycolic acid) copolymer/pluronic polyvinylpyrrolidone	Azithromycin (10 m/1 cm^2^)	$1\;{\mathrm{cm}}^{2}$	Range of 200–550 nm	Yes, albino rabbits	High bioavailability	[56]
						Highly biodegradable and biocompatible	
						Drug release over 10 days	
10	Polycaprolactone (PCL),polyvinyl alcohol (PVA)	Timolol maleate (0.5% *w*/*v*), and dorzolamide hydrochloride (0.2% *w*/*v*)	$1\;\times \;1\;{\mathrm{cm}}^{2}$	Range of 200–400 nm	Yes, New Zealand white albino rabbits	Development of nanofibrous patch for insertion into cul-de-sac for treatment of glaucoma	[57]
						Fibers were uniform and smooth	
						Results showed significant bioadhesion with sustained drug release up to 24 h	
						No ocular irritation with PVA; mild irritation with PLC formulation	
						Significant reduction of intraocular pressure	
11	Chitosan/polyvinyl alcohol (CS/PVA), Eudragit RL100	Ofloxacin (OFX) (0.6% *w*/*v*)	Thickness range: 0.075 ± 0.002 to 0.095 ± 0.002 mm	Average 123 ± 23 to 159 ± 30 nm	Yes, New Zealand white albino rabbits	Two-layer ocular inserts for enhancement of residence time were developed	[58]
						Acceptable morphological and mechanical parameters	
						Release up to 95 h	
						In vivo studies revealed no ocular irritation	
12	Chitosan, polyvinyl alcohol (PVA), polyvinyl pyrrolidone (PVP), Eudragit S100,Zein	Triamcinolone acetonide (1% *w*/*v*)	NA	Range of 120 ± 30 to 172 ± 48 nm	No in vivo studies have been done	Development and evaluation of chitosan-based ocular inserts for sustained drug release	[38]
						Prolonged release was obtained (zero-order kinetics)	

**Table 2:** Ocular inserts formulations and base types.

Drug (Concentration)	Applied Polymer Base	Type of Base (Soluble/Insoluble/Erodible)	Effect/Aim	References
Azithromycin (20% *w*/*w*)	Hydroxypropylmethyl cellulose (HPMC) and Eudragit RL100	Erodible	To prolong release and improve ocular availability	[10]
			Release: over 12 h	
Triamcinolone acetonide (0.5% *w*/*w*)	Poly(1,4-butylene succinate) extended with 1,6-diisocyanatohexane (PBS)	Insoluble	Non-erodible mucoadhesive to sustain release	[34]
			Release of active for 30 days	
Azithromycin (10% *w*/*w*)	Hydroxypropyl methylcellulose (HPMC) or hydroxyethyl cellulose (HEC).	Soluble and erodible	Polymeric inserts to sustain drug release	[59]
			Significantly prolonged release of AZM in rabbit eyes (121 h)	
Cetirizine (7.5% *w*/*v*)	Hydroxypropyl methylcellulose (HPMC) and polyvinyl alcohol (PVA)	Soluble and erodible	To ensure sustained drug release and increased residence time	[60]
			210 min	
Ofloxacin (1.5 and 6% *w*/*w*)	Poly(ethylene oxide) (PEO 400 or PEO 900)	Erodible	To be able to release different drugs of interest in ophthalmology	[61]
Besifloxacin HCl (40 μg/${\mathrm{cm}}^{2}$)	Poly(caprolactone)/polyethylene glycol (PLC/PEG) (2:1)	Erodible	To reduce application frequency and increase patient compliance	[33]
			7 days	
Dorzolamide HCl (0.37% *w*/*v*)	Polyvinyl alcohol, poloxamer 407	Soluble	Comparative result shows that dorzolamide HCL soluble insert was more effective than marketed formulation	[62]
Timolol maleate (TM) (0.5% *w*/*v*)	Hyaluronic acid (HA) and hydroxypropyl methylcellulose (HPMC)	Erodible	To enhance drug retention on ocular surface and potentially improve bioavailability	[63]
Moxifloxacin hydrochloride (5% *w*/*w*)	Eudragit™ FS-100 (FS) and propylene glycol (PG)	Soluble	To provide a once-a-day application as an alternative delivery system in management of bacterial infections	[64]
Gatifloxacin (2.4 mg/78.5 mm^2^)	Thiolated sodium alginate (TSA), sodium alginate (SA), and acrylates: ERL:ERS (75:25)	Erodible	Inserts for twice-a-day therapy with gatifloxacin	[65]
Dexamethasone (1%, 5% and 10% *w*/*w*)	Polylactic acid (PLA) and poly-vinyl alcohol (PVA)	Erodible	Nanofibrous inserts were better than solvent cast inserts and could be utilized as a potential delivery system for treating anterior segment ocular diseases	[52]
Curcumin (1% *w*/*w*)	Carboxymethylcellulose (CMC), polyvinyl alcohol, hydroxypropyl methylcellulose	Soluble	Developed inserts demonstrated acceptable ocular tolerability, enhanced corneal permeability, and sustained release	[66]
Lysozyme (3% *w*/*w*)	Hydroxypropyl methylcellulose (HPMC)	Erodible	Novel drug delivery system (DDS) with sustained release properties was developed to allow ocular protein delivery	[67]
Gentamicin sulfate (25.0% *w*/*w)*	Mixture of hydroxypropyl cellulose, ethyl cellulose, poly(acrylic) acid	Soluble	Inserts ensured effective gentamicin levels over 72 h	[68]
Indomethacin (IN; 10% *w*/*w*), prednisolone sodium phosphate (PSP; 10% *w*/*w*), ciprofloxacin hydrochloride (CIP; 10% *w*/*w*)	Polyethylene oxide (PEO) N10	Erodible	Noninvasive ocular inserts for posterior segment	[69]
			Trans-membrane flux of IN, prednisolone sodium phosphate, and ciprofloxacin hydrochloride was enhanced by ~3.5, ~3.6, and ~2.9-fold, respectively	
			Ocular inserts generated significantly higher drug levels in all ocular tissues, including retina-choroid, compared with control formulations	
Valacyclovir HCl (20% *w*/*w*)	HPC and HPMC	Soluble and erodible	Ocular inserts showed improved flux compared with control formulation	[70]
			Ocular inserts dissolved completely within 8 h	
Fluorescein (3% *w*/*w*), lysozyme (3% *w*/*w*), albumin (3% *w*/*w*)	HPMC	Erodible	Ophthalmic inserts with sustained release properties as carriers for thermo-labile therapeutics	[71]
Gentamicin sulfate (25.0% *w*/*w*), dexamethasone phosphate (5.0 and 25% *w*/*w*)	Hydroxypropyl cellulose (HPC)	Soluble	New system ensures concomitant release of drugs during first 10 h of treatment, followed by adequate concentration of gentamicin sulfate	[72]
Ketorolac tromethamine (KT; 1% *w*/*v*)	Eudragit S100 and Zein	Erodible	Ocular delivery system using electrospun nanofibers as candidate insert for delivery of triamcinolone acetonide	[38]
			To improve bioavailability	
Ofloxacin (3–9% *w*/*w*)	Chitosan/polyvinyl alcohol (CS/PVA), Eudragit RL100.	Erodible	To enhance ocular residence time of ofloxacin	[58]
			Sustained release pattern up to 96 h	
Brimonidine tartrate (≈10–30% *w*/*w*)	PEO with Eudragit (RL 100/RS 100)	Erodible	To design mucoadhesive and extended release ocular inserts; up to 24 h	[73]
Linezolid (LNZ; 0.2% *w*/*v*)	Modified sodium alginate-grafted poly (butyl methacrylate) and sodium alginate-grafted poly (lauryl methacrylate)	Erodible	Polymeric thin films with increased ocular residence time and sustained drug release capacity	[74]

**Table 3:** Summary of ocular insert patents studies (2011–2021).

Insert Base	Targeted Diseases	Model Drug Used for Study	Applicant /Manufacturer/Assignee	Patent Number	Publication Date	Reference
Biodegradable, hydrophobic polymer	Tissue regeneration, glaucoma, infections on eye surface (all are possible)	Travoprost	Integral Biosystems LLC	AU 2019250153 A1	31 October 2019	[75]
Biodegradable, hydrophobic, and/or amphiphilic polymer	Eye infections	Azithromycin	Zewail City of Science and Technology, Egypt	GB 2570113 A	17 July 2019	[76]
Hydrophilic, biodegradable, bioabsorbable, or bioerodible polymer	Glaucoma	Timolol maleate	Valeant International (Barbados) SRL	US2012215184A1	23 August 2012	[77]
Thermoplastic polymer such as acrylonitrile butadiene styrene and ethylene-vinyl acetate	Glaucoma	Bimatoprost	ForSight VISION5 Inc. (US)	US2016022695A1	28 January 2016	[78]
Polyvinyl-alcohol (PVA)	Bacterial infections (tuberculosis (TB))	Rifampicin	Tongling Wutongshu Agricultural Dev. Co. Ltd.	CN104116722A	29 October 2014	[79]
Polymer such as poly-lactic/glycolic acid	Ocular inflammation associated with inflammatory diseases or following ocular surgery	Steroids	Interface Biologics Inc. (CA)	WO2019148291A1	08 August 2019	[80]
Arylborono-containing hydrophilic copolymer	Anterior and posterior segment diseases	Optional	Alcon Inc. (CH)	US2021077385A1	18 March 2021	[81]
Hydroxypropyl methylcellulose, polyvinylpyrrolidone, sodium carboxymethyl cellulose, and gelatin	Myopia	Atropine sulfate	Univ. Shenyang Pharmaceutical	CN111358771A	07 July 2020	[82]
Hydroxypropylcellulose and polyethylene oxide block copolymer	Glaucoma	Timolol maleate	Univ. Witwatersrand JHB (ZA)	WO2014041485A1	20 March 2014	[83]
Thermoplastic polymer such as acrylonitrile butadiene styrene and thermosetting polymer such as silicone and polyesters	Glaucoma	Bimatoprost	ForSight VISION5 Inc. (US)	US2016296532A1	13 October 2016	[84]
Polycaprolactone/polyethelyneglycol	Bacterial infections	Moxifloxacin	Univ. De Coimbra (PT)	WO2017137934A1	17 August 2017	[85]
Poly-vinyl-alcohol	Fungal infections	Voriconazole	Univ. Zhejiang	CN105726517A	06 July 2016	[86]
Hyaluronic acid, polyvinyl-pyrrolidone, hydroxypropyl guar, and polyethyleneglycol	Dry eye	-	Alcon Inc. (CH)	WO2021116907A1 WO2020222195A1 US2021169781A1	17 June 2021 05 November 2020 10 June 2021	[87,88,89]

## Data Availability

Not applicable.
